# Ferroptosis: A New Regulatory Mechanism in Osteoporosis

**DOI:** 10.1155/2022/2634431

**Published:** 2022-01-17

**Authors:** Pan Liu, Wenzhao Wang, Zheng Li, Yao Li, Xiaoping Yu, Ji Tu, Zhengdong Zhang

**Affiliations:** ^1^School of Clinical Medicine, Chengdu Medical College, Chengdu 610500, China; ^2^The First Affiliated Hospital of Chengdu Medical College, Chengdu 610500, China; ^3^Department of Orthopedics, West China Hospital of Sichuan University, Chengdu 610041, China; ^4^People's Hospital of Jiulongpo District, Chongqing 400050, China; ^5^School of Public Health, Chengdu Medical College, Chengdu 610500, China; ^6^Basic Medical College of Chengdu University, Chengdu 610500, China; ^7^Spine Labs, St. George & Sutherland Clinical School, University of New South Wales, Sydney, Australia; ^8^Department of Orthopedics, The First Affiliated Hospital of Chengdu Medical College, Chengdu 610500, China

## Abstract

Osteoporosis can be caused by a multitude of factors and is defined by a decrease in bone density and mass caused by the destruction of bone microstructure, resulting in increased bone brittleness. Thus, it is a systemic bone disease in which patients are prone to fracture. The role of ferroptosis in the pathogenesis of osteoporosis has become a topic of growing interest. In this review, we discuss the cell morphology, basic mechanisms of ferroptosis, the relationship between ferroptosis and osteoclasts and osteoblasts, as well as the relationship between ferroptosis and diabetic osteoporosis, steroid-induced osteoporosis, and postmenopausal osteoporosis. Emerging biomedical research has provided new insights into the roles of ferroptosis and osteoporosis, such as in cellular function, signaling pathways, drug inhibition, and gene silencing. The pathophysiology and mechanism of ferroptosis and osteoporosis need to be further studied and elucidated to broaden our understanding of iron metabolism and immune regulation. Studies using animal models of osteoporosis in vivo and cell models in vitro will help clarify the relationship between ferroptosis and osteoporosis and provide research ideas for the elucidation of new mechanisms and development of new technologies and new drugs for the treatment of osteoporosis in the future.

## 1. Introduction

Cell death includes apoptosis, pyroptosis, necrosis, autophagy, ferroptosis, and other death mechanisms. Before the concept of ferroptosis was revealed, one study showed that the regulation of iron metabolism and the maintenance of iron homeostasis have indispensable biological roles in the human pathophysiological process. In 2003, Dolma et al. [1] found that the small molecule erastin can induce RAS mutations in tumor cells, leading to cell death in a manner different from traditional apoptosis. In 2008, using high-throughput small-molecule screening technology, it was found that Ras selective lethal small molecules could kill human foreskin fibroblasts (BJeLR) in a nonapoptotic manner [2]; however, neither apoptosis inhibitors nor necrostatin inhibitors (Necrostatin-1) [3] could reverse cell death induced by erastin and RSLs. In contrast, the antioxidant vitamin E and the iron-chelating agent deferoxamine mesylate (DFO) can inhibit cell death [4], indicating that ferroptosis is an iron-dependent cell death process. In 2012, Dixon et al. named this process erastin-induced cell death with distinct morphological, genetic, and biochemical characteristics [5].

Iron, an essential trace element for humans and a necessary substance for life, plays a vital role in many biochemical processes, including oxygen transport, enzymatic reactions, and immune reactions. With accumulating research, various physiological and pathological processes, such as tumor, Parkinson's disease, atherosclerosis, viral infection, osteoporosis, immune response, and ischemia-reperfusion injury, have been found to be related to ferroptosis [6–8], and ferroptosis is expected to become a new research direction for disease treatment.

Interestingly, a growing number of studies have reported a relationship between ferroptosis and osteoporosis. Here, we summarize the basic pathological features of ferroptosis and the relationship between ferroptosis and osteoclasts and osteoblasts. We also summarize the relationship between ferroptosis and osteoporosis and show how ferroptosis regulates diabetic osteoporosis (DOP), glucocorticoid-induced osteoporosis (GIOP), and postmenopausal osteoporosis (PMOP).

## 2. Morphological Characteristics of Ferroptosis

Ferroptosis was delineated as a type of regulated cell death (RCD) by the Nomenclature Committee on Cell Death in 2018. It is initiated by oxidative perturbations in the intracellular microenvironment, which are constitutively controlled by glutathione peroxidase 4 (GPX4) and can be inhibited by iron-chelating agents and lipophilic antioxidants [9]. Ferroptosis is distinguished from apoptosis [10], necrosis [11], autophagy [12], and pyroptosis [13] by a varying set of morphological characteristics, inducing factors, and regulatory pathways.

Cancer cells undergoing ferroptosis are generally round, small, and scattered [1]. In human prepuce fibroblast BJeLR cells treated with erastin, several effects were observed: mitochondria atrophied and decreased in number, membrane density increased, the normal structure of mitochondrial cristae was destroyed, nuclear size was normal but lacked chromatin aggregation, and cell membranes were blistered without rupturing [5, 14–16]. These morphological features can help to distinguish ferroptosis from other modes of cell death, such as apoptosis, necrosis, pyroptosis, and autophagy.

## 3. Mechanisms and Regulation of Ferroptosis

### 3.1. Iron Metabolism and Ferroptosis

Iron is involved in the synthesis of various important proteases and is an essential element in the life activities of the body [17, 18]. Iron overload caused by abnormal iron metabolism is one of the main characteristics of ferroptosis. Binding of ferric acid to transferrin is the main mechanism for circulation of iron in the bloodstream. Circulating iron enters cells by binding to transferrin receptor 1 (TFR1) on the cell membrane, where the six-transmembrane epithelial antigen of prostate 3 (STEAP3) reduces ferric iron to ferrous iron. Finally, divalent metal transporter 1 (DMT1) releases divalent iron into the labile iron pool (LIP) in the cytoplasm. LIP enables the active uptake of free iron in the cytoplasm, as well as recovery of iron in ferritin and mitochondria, and large quantities of LIP are present in lysosomes [19]. Lysosomes are therefore considered the main organelles responsible for cellular ferroptosis [20]. Excess bivalent iron is transported extracellularly by ferroportin 1 (FPN1) and stored in ferritin heavy chain 1 and ferritin light chain 1 (FTL1) [21, 22].

Under physiological conditions, ferritin provides a strong buffer that regulates physiological responses to iron deficiency and excess, maintaining homeostasis [23]. Under pathological conditions, iron overload can induce ferroptosis by producing ROS through Fenton and Haber–Weiss reactions [24, 25]. Studies have shown that DFO, an iron chelator, can inhibit ferroptosis caused by intracellular iron overload [26, 27]. In addition, a high iron diet can lead to serious heart damage, and the ferroptosis inhibitor ferrostatin-1 mitigates the damage caused by ferroptosis [28]. Under physiological conditions, mitochondrial ferritin (FtMt) regulates the free iron content in the mitochondria and maintains normal mitochondrial iron metabolism. FtMt overexpression can reverse erastin-induced ferroptosis both in vivo and in vitro [29, 30]. In neurological diseases, mitochondrial transferrin mitoferrin 1/2 (Mfrn1/2) on the inner mitochondrial membrane is found to be destroyed, resulting in abnormal iron metabolism in the mitochondria [31].

Iron metabolism has been implicated in the occurrence and development of ferroptosis. However, the role of systemic iron regulation in the cellular role of iron deposits and ferritinophagy remains elusive. Whether systemic iron levels fully determine the effect of ferroptosis on the disease remains to be clarified.

### 3.2. Ferroptosis Mediated by P53

P53 is an important tumor suppressor gene with important roles in cell cycle inhibition, apoptosis, tumorigenesis, and aging [32–34]. mRNA and protein expression levels of SLC7A11 were significantly decreased after the upregulation of p53 gene expression, which confirmed that SLC7A11 is a new target of the p53 gene [35, 36]. p53-silenced H1299 cells were treated with ROS, and there was no change in cell activity. In contrast, cells with activated p53 that were treated with ROS had a 90% death rate. After adding the ferroptosis inhibitor, ferrostatin-1, the cell death rate decreased by approximately 40%, indicating that p53 can induce ferroptosis [37]. Recent studies have shown that P53 can inhibit the uptake of cystine by system xc^−^ by downregulating the expression of the SLC7A11 subunit, resulting in a decrease in cystine-dependent glutathione peroxidase activity and cell antioxidant capacity and an increase in lipid ROS, leading to cellular ferroptosis [38].

### 3.3. Voltage-Dependent Anion Channels with Ferroptosis

Mitochondria, the main regulators of oxidative phosphorylation, play an important role in oxidative stress and are major producers of ROS [39, 40]. Iron can reach the mitochondrial matrix by passing through the outer mitochondrial membrane and inner mitochondrial membrane, subsequently regulating the physiological functions of important organelles in the mitochondria [41]. Mitochondria also play an important role in the regulation of ferroptosis [42]. Under erastin induction, the voltage-dependent anion channel protein 2/3 (VDAC2/3) on the outer mitochondrial membrane is opened, leading to iron accumulation in the mitochondria. However, the detailed mechanism underlying the role of erastin and VDAC2/3 is still being explored [43].

### 3.4. Ferroptosis Induced by Inhibition of GPX4 and Cysteine-Glutamate Transporter Receptors (System xc^−^)

GPX4, an important specific marker of ferroptosis [44], can reduce lipid peroxides to lipid alcohols and hydrogen peroxide to water during ferroptosis [45]. Both GPX4 knockout and the use of the small molecule inhibitor RSL3 antagonism against GPX4 could effectively induce ferroptosis [46–49]. L-glutathione (GSH) is composed of glycine, glutamate, and cysteine and is an important antioxidant in the oxidative stress response, widely present in cells in the form of reduced GSH and oxidized glutathione (GSSG) [50]. The degradation of lipid peroxides by GPX4 requires GSH for the provision of electrons to complete the process [20]. GSH synthesis requires intracellular uptake of cysteine, which is mediated by the sodium-dependent system xc^–^ (also named the cystine/glutamate antiporter), a disulfide-linked heterodimer composed of a heavy chain (4F2hc, gene name SLC3A2), and a light chain (xCT, gene name SLC7A11). The sodium-dependent system xc^–^ transports extracellular cystine into the cell and further converts it to cysteine, which is then used in GSH biosynthesis [5, 51–55]. Studies have shown that selective inhibition of system xc^−^ leads to a decrease in intracellular GSH, which aggravates ROS accumulation and eventually leads to ferroptosis [56, 57]. Although a regulatory mechanism between GXP4 and ferroptosis is known to exist, the roles of GXP4 in different RCD and the mechanism of information transduction pathways remain unclear.

### 3.5. Ferroptosis Mediated by Lipid Peroxidation

Lipid peroxidation is another key factor in ferroptosis. Recent studies [58] have shown that lipid peroxides can destroy the stability of the lipid bilayer, causing disintegration of the cell membrane. Lipidomics analyses have indicated that both AA and adrenic acid containing phosphatidyl ethanolamine are lipid products of ferroptosis, which can undergo spontaneous peroxidation in the presence of hydroxyl radicals (produced by the Fenton reaction between redox-active iron divalent and hydrogen peroxide) [46, 59, 60]. Polyunsaturated fatty acids (PUFAs) are prone to lipid peroxidation, owing to the presence of highly active hydrogen atoms in methylene bridges. Hydroxyl radicals can directly interact with PUFAs in membrane phospholipids through chain reactions to form lipid peroxides, which attack the cytomembrane and trigger morphological changes in ferroptosis [61, 62]. Derivatives resulting from the decomposition of lipid peroxides, including 4-hydroxynonenal (4-HNE) and malondialdehyde (MDA), can react with nucleic acids and proteins, leading to further cell destruction [63, 64]. These derivatives can also be used as important molecular markers for the detection of ferroptosis and lipid peroxidation. In addition, divalent iron can be used as a cofactor of lipoxygenase (LOX) to catalyze lipid peroxidation of PUFAs [65]. Recent studies have shown [62] that both lysophosphatidylcholine acyltransferase 3 (LPCAT3) and ACSL4 are involved in lipid peroxidation of membrane PUFAs, and they serve as important molecular markers of ferroptosis.

**Figure 1:**
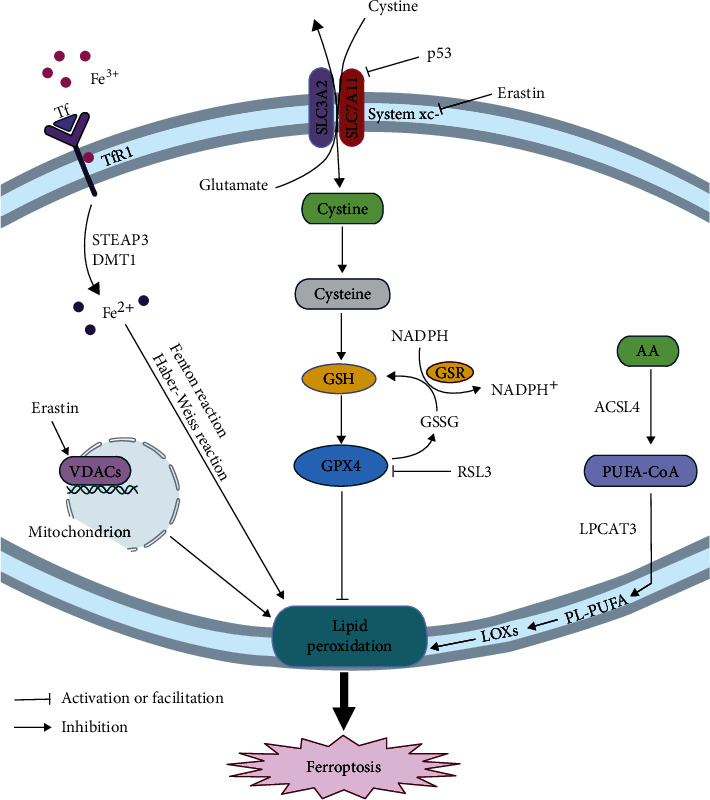
The mechanism of ferroptosis. Circulating iron enters cells by binding to TFR1 on the cell membrane, where the STEAP3 reduces ferric iron to ferrous iron. DMT1 releases the divalent iron into a labile iron pool in the cytoplasm, and iron overload can induce ferroptosis by producing ROS through the Fenton and Haber–Weiss reactions. The sodium-dependent system xc^–^ transports extracellular cystine into the cell and further converts it to cysteine. The selective inhibitor of system xc^−^ leads to a decrease in intracellular GSH, which aggravates ROS accumulation and eventually leads to ferroptosis; P53 can inhibit the uptake of cystine by system xc^−^ via downregulating the expression of the SLC7A11 subunit, resulting in a decrease in cystine-dependent glutathione peroxidase activity and cell antioxidant capacity and an increase in lipid ROS, leading to ferroptosis of cells; RSL3 can induce ferroptosis by antagonizing GPX4; Hydroxyl radicals can directly interact with PUFAs in membrane phospholipids through chain reactions to form lipid peroxides, inducing ferroptosis.

In the upper panel, we can see that ferroptosis involves multiple signaling pathways and their regulators (Figure 1). Understanding these signaling molecules and their transduction pathways is of great significance in the pathophysiology of ferroptosis.

### 3.6. Ferroptosis with Osteoclasts and Osteoblasts

Over the last two decades, the relationship between iron and osteoporosis has attracted increasing attention. Studies have reported that disorders of iron metabolism, including iron deficiency and iron overload, can lead to osteoporosis [66–70]. The homeostasis and integrity of bone tissue are maintained by maintaining a balance between osteoclastic and osteogenic activities, and the remodeling process of bone tissue is a continuous cycle. Osteoclasts mainly play the role of bone resorption, whereas osteoblasts mainly play the role of bone reconstruction, such as the formation, mineralization, and secretion of osteocytes. They mutually restrict and balance the metabolism of bone tissues [71, 72]. Song et al. found that FA complementation group D2 (FANCD2) suppresses erastin-induced ferroptosis in bone mesenchymal stem cells (BMSCs), and FANCD2 reduces iron accumulation and lipid peroxidation in ferroptosis [73]. This is due to the multidirectional differentiation potential of BMSCs. These results suggest that ferroptosis may also occur during the targeted differentiation of BMSCs under certain circumstances.

### 3.7. Ferroptosis May Occur in Osteoclasts

Osteoclasts are large multinucleated cells formed by the fusion of mononuclear macrophage lineage or BMSCs by the inductive form of receptor activator of nuclear factor-kappa B ligand (RANKL) and perform the function of bone resorption. Iron ions can promote osteoclast differentiation and bone resorption by producing ROS [74]. The iron chelator DFO inhibits osteoclast formation in vitro [75]. Liu et al. found the iron-starvation response and ferritinophagy under normoxia in the process of osteoclast differentiation confirmed the involvement of ferroptosis; following RANKL stimulation, MDA and prostaglandin endoperoxide synthase 2 (PTGS2) gene expression in bone marrow-derived macrophages (BMDMs) were increased, GSH and iron levels in the culture medium supernatant decreased, and iron accumulation in mitochondria was observed [76].

### 3.8. Ferroptosis May Occur in Osteoblasts

Osteoblasts play an important role in bone regeneration and play a leading role in the synthesis, secretion, and mineralization of the bone matrix [77]. Previous studies have shown that the inhibitory effect of iron on the osteogenic differentiation of MSCs is proposed, and iron overload in mice is associated with increased ferritin and decreased RUNX family transcription factor 2 (RUNX2) levels in compact bone osteoprogenitor cells [69]. A high dose of dexamethasone (10 *μ*M dexamethasone) may activate osteoblasts to induce ferroptosis by downregulating GPX4 and system xc^−^ [78]. Subsequent studies found that GPX4 was significantly reduced, ROS levels were increased in MC3T3 cells induced by high glucose, and mitochondria were generally smaller and less tubular, with a darker-stained membrane with distinctly disrupted inner membrane folding. In addition, the ability of MC3T3 to differentiate into osteoblasts and the formation of mineralized nodules was decreased in a high glucose environment, and similar phenomena were observed in osteoblasts in mice [79, 80].

Based on the results of the above studies, we hypothesized that ferroptosis of osteoclasts would reduce the occurrence of bone resorption, while ferroptosis of osteoblasts would lead to reduced bone formation.

## 4. Potential Relationship between Ferroptosis and Osteoporosis

Osteoporosis is a metabolic bone disease, involving an imbalance between the bone resorptive functions of osteoclasts and bone forming functions of osteoblasts. This imbalance leads to loss of bone mass and strength, resulting in the increased risk of fragility fractures and a progressive decrease in healing ability following fractures [81–83]. Global rates of hip fractures, vertebral fractures, and wrist fractures caused by osteoporosis have increased, emerging as a major global public health problem [84] with approximately 8.9 million people worldwide experiencing osteoporotic fractures each year [85]. It is estimated that by 2050, hip fractures in elderly men will increase by 310%, and hip fractures in elderly women will increase by 240% [86]. Preventing and effectively treating the occurrence and development of osteoporosis are therefore an urgent priority in global health.

### 4.1. Ferroptosis and DOP

Approximately 1 in 11 adults worldwide have diabetes, and 90% have type 2 diabetes [87]. Diabetes mellitus is often associated with osteoporosis [88] that this is often associated to multiple factors. It has been suggested that the factors contributing to reduced bone formation include oxidative stress caused by high blood sugar and accumulation of advanced glycation end products (AGEs) in collagen [88, 89]. Other studies suggest that low concentrations of insulin and insulin-like growth factor 1 (IGF-1) may affect osteogenic activity and lead to osteoporosis [90]. Antidiabetic drugs, such as thiazolidinedione, have been shown to negatively affect bone metabolism and fracture risk [91, 92]. However, the detailed pathological mechanism is not fully understood and is still being explored. In addition, patients with diabetes often have complications such as vision loss and neuropathy, which can increase the risk of falls and fractures. An in-depth study of the pathological mechanism of DOP would help to improve the prediction of DOP risk, as well as enable timely and reasonable prevention and treatment of brittle fractures caused by osteoporosis.

Iron metabolism is often disturbed in patients with diabetes [93–95]. Iron is also a strong oxidant that can promote the production of many reactive oxygen free radicals. Indicators of iron metabolism (transferrin, ferritin, hepcidin, transferrin receptor, etc.) can directly or indirectly affect the occurrence and development of type 2 diabetes [94]. Ferroptosis results in the production of abundant ROS through the Fenton reaction, which causes the accumulation of lipid peroxides and cell damage [96]. Wang et al. [80] detected the expression of FtMt and the occurrence of ferroptosis in the bone tissue of a (T2DOP) rat model of type 2 diabetes. They found that overexpression of FtMt reduced oxidative stress induced by excess iron under high glucose conditions to inhibit the occurrence of ferroptosis in osteoblasts, while the silencing of FtMt induced mitochondrial autophagy of T2DOP through the ROS/PINK1/Parkin signaling pathway. This suggests that FtMt may be a potential target for the treatment of T2DOP. Meanwhile, it was found that ferroptosis of osteoblasts increased following mitochondrial activation by carbonyl cyanide-M-chlorophenyl-hydrazine (CCCP, a mitochondrial agonist). Additionally, several other effects were observed: decreased expression of GPX4, osteocalcin (OCN), alkaline phosphatase (ALP), and osteoprotegerin (OPG), decreased mineralized nodules, increased ROS levels, and increased lipid peroxide. In contrast, treatment of the CCCP group with ferroptosis inhibitors was able to rescue ferroptosis. Ma et al. [79] found that high glucose induced ferroptosis in the bone tissue of a T2DOP rat model by increasing the consumption of ROS/lipid peroxidation/GSH. More importantly, melatonin (N-acetyl-5-methoxytryptamine) significantly reduced the level of ferroptosis by activating the Nrf2/HO-1 signaling pathway in vivo and in vitro and improved the osteogenic ability of MC3T3-E1 cells. Based on these studies, we speculated that the occurrence of T2DOP was correlated with iron homeostasis imbalance and ferroptosis in osteoblasts. Nonetheless, the detailed mechanisms require further investigation.

### 4.2. Ferroptosis and GIOP

Ferroptosis is a recently discovered form of cell death characterized by lipid peroxidation caused by the downregulation of GPX4 and system xc^−^. It is involved in GIOP. Yang et al. [97] found that high-dose and long-term use of steroid hormones can alter antioxidant capacity, reduce the activity and function of osteoblasts, and lead to osteoporosis and osteonecrosis. Endothelial cell-secreted exosomes (EC-Exos) are important mediators of cell-to-cell communication and are involved in many physiological and pathological processes. By inhibiting ferritin-phagocytosis-dependent ferroptosis, EC-Exos reversed the inhibitory effect of glucocorticoid-induced osteoblasts on osteogenesis. Lu et al. [78] established a GIOP model with a high dose of dexamethasone and found that high-dose dexamethasone (10 *μ*M) can induce ferroptosis of osteoblasts, possibly by downregulating GPX4 and system xc^−^. KEGG-based gene set enrichment analysis was performed to demonstrate the activation of the ferroptosis pathway. Extracellular vesicles extracted from bone marrow-derived endothelial progenitor cells inhibited activation of the ferroptosis pathway by restoring GPX4 and system xc^−^. The changes in the expression of ferroptosis markers, such as SLC3A2, SLC7A11, and GPX4, were further confirmed using RNA-seq. EPC-EVS reversed dexamethasone-induced changes in cysteine and oxidative damage markers, such as MDA, GSH, and glutathione disulfide (GSSG), and improved skeletal parameters in mice. EPC-EVS reversed dexamethasone-induced changes in cysteine and oxidative damage markers, such as MDA, GSH, and GSSG, and improved skeletal parameters in mice. They suggested that EPC-EVS prevents glucocorticoid-induced osteoporosis in mice by inhibiting the ferroptotic pathway of osteoblasts. However, further research is needed to elucidate the ferroptosis mechanisms associated with GIOP.

### 4.3. Ferroptosis and PMOP

PMOP is caused by estrogen deficiency in postmenopausal women. Estrogen deficiency is associated with insufficient differentiation of osteoblasts and increased activity of osteoclasts, ultimately leading to decreased bone mass and increased bone fragility [98]. A significant association between low serum iron levels and PMOP has been reported [66, 99]. Abraham et al. [100] found that dietary iron (or related factors) may have a protective effect against bone loss in the postmenopausal spine. Ni et al. [76] found a correlation between ferroptosis and RANKL-induced osteoclast differentiation and iron-starvation response, and ferritinophagy promoted the ferroptosis of osteoclasts induced by RANKL. In vivo, the HIF-1*α*-specific inhibitor 2-methoxyestradiol (2ME2) was found to prevent bone loss in OVX mice. The authors, therefore, proposed that the induction of ferroptosis of osteoclasts by targeting HIF-1*α* and ferritin could be an alternative treatment for osteoporosis.

**Figure 2:**
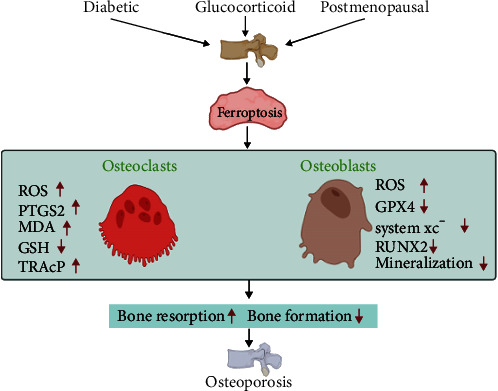
Diabetic, glucocorticoid, and postmenopausal induce ferroptosis in osteoclasts and osteoblasts. The subsequent loss of bone mass contributes to osteoporosis. RUNX2: Runt-related transcription factor 2; TRAcP: tartrate resistance acid phosphatase.

Together, these findings show that patients with osteoporosis often experience iron metabolism disorders, oxidative stress, and lipid peroxidation, leading to ferroptosis (Figure 2). This suggests that the regulation of ferroptosis of osteoclasts or osteoblasts may provide a potential therapeutic strategy for osteoporosis (Table 1).

## 5. Conclusion

Owing to consistent research efforts, osteoporosis has developed from a highly disabling disease to a disease that can be managed. Existing drugs and biologics marketed to treat osteoporosis have some clinical efficacy, but are associated with side effects including cancer, osteonecrosis of the jaw, and adverse effects on liver and kidney function. Improved antiosteoporosis treatment is therefore a significant priority for medical researchers and clinicians.

In this review, we have summarized the cell morphology, cell characteristics, and pathogenesis of ferroptosis, as well as the relationship between ferroptosis and osteoporosis. Ferroptosis is an iron-dependent nonapoptotic form of RCD, which is closely related to the pathophysiological processes of various human diseases. It is accompanied by disorders of iron metabolism and energy metabolism, upregulation of inflammation and oxidative stress, and functional impairment of important organelles in cells.

Elucidating the molecular mechanisms of ferroptosis and osteoporosis can provide substantial insights into the field of bone metabolism and immunity, enabling the discovery of new therapeutics with fewer side effect for the prevention and treatment of osteoporosis. At present, these encouraging research results have generated great interest in further exploring the mechanisms underlying iron-dependent cell death and osteoporosis. The interaction and crosstalk among osteoclasts, osteoblasts, and osteocytes should be considered in the treatment of osteoporosis. Research on ferroptosis is still at a relatively early stage, and the specific mechanism, nodal molecules, and related signaling pathways of ferroptosis remain unclear. Further study is required to explore these newly discovered mechanisms, their associated signaling pathways and molecular targets, to develop effective treatment methods. The use of osteoporosis models in castrated mice, diabetic mice, and aging mice will help to determine the relationship between osteoporosis and ferroptosis and guide research for further understanding and effectively treating osteoporosis.

## Figures and Tables

**Table 1:** Interventions and reagents targeting ferroptosis for osteoporosis.

Intervention methods or reagents	Mechanism	Effects on cells	Reference
2ME2 (2-methoxyestradiol)	Targeting HIF-1*α* and ferritin	Inducing the ferroptosis of osteoclasts	[76]
EPC-EVs	Restoring GPX4 and system xc^−^ levels	Inhibiting ferroptotic pathway of osteoblasts	[78]
Melatonin	Activating the Nrf2/ho-1 signaling	Reducing ferroptosis in MC3T3-E1	[79]
Silencing FtMt	Inducing mitophagy via ROS/PINK1/Parkin pathway	Inhibiting ferroptosis of osteoblasts	[80]
CCCP (mitophagy agonist)	Activating mitochondria	Promoting ferroptosis of osteoblasts	[80]
EC-exos	Inhibiting ferritin-phagocytosis-dependent ferroptosis	Reversing the inhibitory effect of glucocorticoid on osteoblasts	[97]
